# A Case Report of Late Diagnosis of Bilateral Choanal Atresia

**DOI:** 10.7759/cureus.17530

**Published:** 2021-08-28

**Authors:** Rahmah A ALaryani, Jaber Alshammari, Wala S Alshiha

**Affiliations:** 1 Ear Nose and Throat (ENT), Prince Sultan Military Medical City (PSMMC), Riyadh, SAU; 2 Division of Otolaryngology Head & Neck Surgery, King Abdullah Specialized Children Hospital (KASCH) King Abdulaziz Medical City (KAMC) National Guard Health Affairs (NGHA), Riyadh, SAU; 3 Division of Pediatric Otolaryngology, Head & Neck Surgery, Prince Sultan Military Medical City (PSMMC), Riyadh, SAU

**Keywords:** diagnosis, bilateral, choanal atresia, late, congenital

## Abstract

Choanal atresia (CA) is a congenital closure of the posterior nasal choanae. The closure can be unilateral or bilateral. Since the first report of CA, there have been controversies regarding its pathogenesis and the effectiveness of surgical approaches. The few cases reported in the literature were not diagnosed immediately after birth. We present a case of delayed presentation of CA. The patient was born pre-term (35 weeks) by cesarean section. He was diagnosed with bilateral CA by history, physical examination, endoscopic and radiologicalimages at five months of age.

Congenital bilateral CA is rarely discovered in neonatal patients after one week of age, therefore few such cases have been reported in the literature. Nasal endoscopy and computed tomography help diagnose CA before planning surgery. Several surgical approaches for repairing congenital CA have been reported, including the transnasal and transpalatal routes. Endoscopic transnasal choanoplasty is currently the preferred approach.

Though CA is a rare congenital malformation, there are cases with a delayed diagnoses reported in the literature. These findings raise the question of whether all newborns are obligate nasal breathers. Although rare, bilateral CA should be suspected in infants who exhibit difficulty with feeding and bilateral nasal obstruction, as in such cases it is impossible to feed and breathe simultaneously.

## Introduction

Choanal atresia (CA) is a congenital obstruction of the posterior choanae of the nasal cavity [1]. Anatomically, it results from a medialized lateral pterygoid plate and enlarged vomer [2]. Congenital CA was first described by Johann Roderer in 1755 during the clinical evaluation of a newborn with total choanal closure [3]. In 1854, Emmert reported the first successful operation through the transnasal approach for treating congenital CA in a seven-year-old child [3]. The incidence of this malformation ranges from one in 5,000 to one in 8,000 live births [4-5].

It is common for unilateral CA to present later in life, whereas bilateral CA is usually diagnosed in the first week because it is considered being incompatible with life and is rarely seen several weeks or months after the baby is born. An extensive search for the late diagnosis of bilateral congenital choanal atresia, revealed, to our knowledge, only a few reported cases to date. In this study, we present a case of delayed congenital CA in a five-month-old pre-term baby, with a review of the clinical/radiological features and patient management to help otolaryngologists in their practice.

## Case presentation

A five-month-old pre-term male neonate was born by cesarean section at 35 gestational weeks due to a macrocephaly. The birth weight was 2.8 kg. He was transferred to the neonatal intensive unit (NICU) for approximately one month and intubated for two weeks and then moved to the nursery for observation for two months without the involvement of Otorhinolaryngology. The patient had a history of paramedian facial cleft, craniofacial anomalies (occipital scalp defect, midface hypoplasia, absence of the right nasal ala, incomplete cleft lip, and right medial epicanthal fold defect), kyphoscoliosis, central hypotonia, congenital heart disease, and recurrent history of upper respiratory tract infections. He underwent ventriculoperitoneal shunting for the treatment of hydrocephalus.

The patient presented at the emergency department of King Abdulaziz Medical City with two episodes of apnea in two days, with cyanosis around the mouth for less than one minute (both episodes resolved spontaneously), dyspnea, poor feeding, decreased activity, fever, and increased nasal secretions. He was admitted to the pediatric intensive care unit (PICU) as a case of bronchiolitis to rule out sepsis. He was then referred to the otorhinolaryngology clinic for difficulty breathing and continuous mucoid nasal discharge.

**Figure 1:**
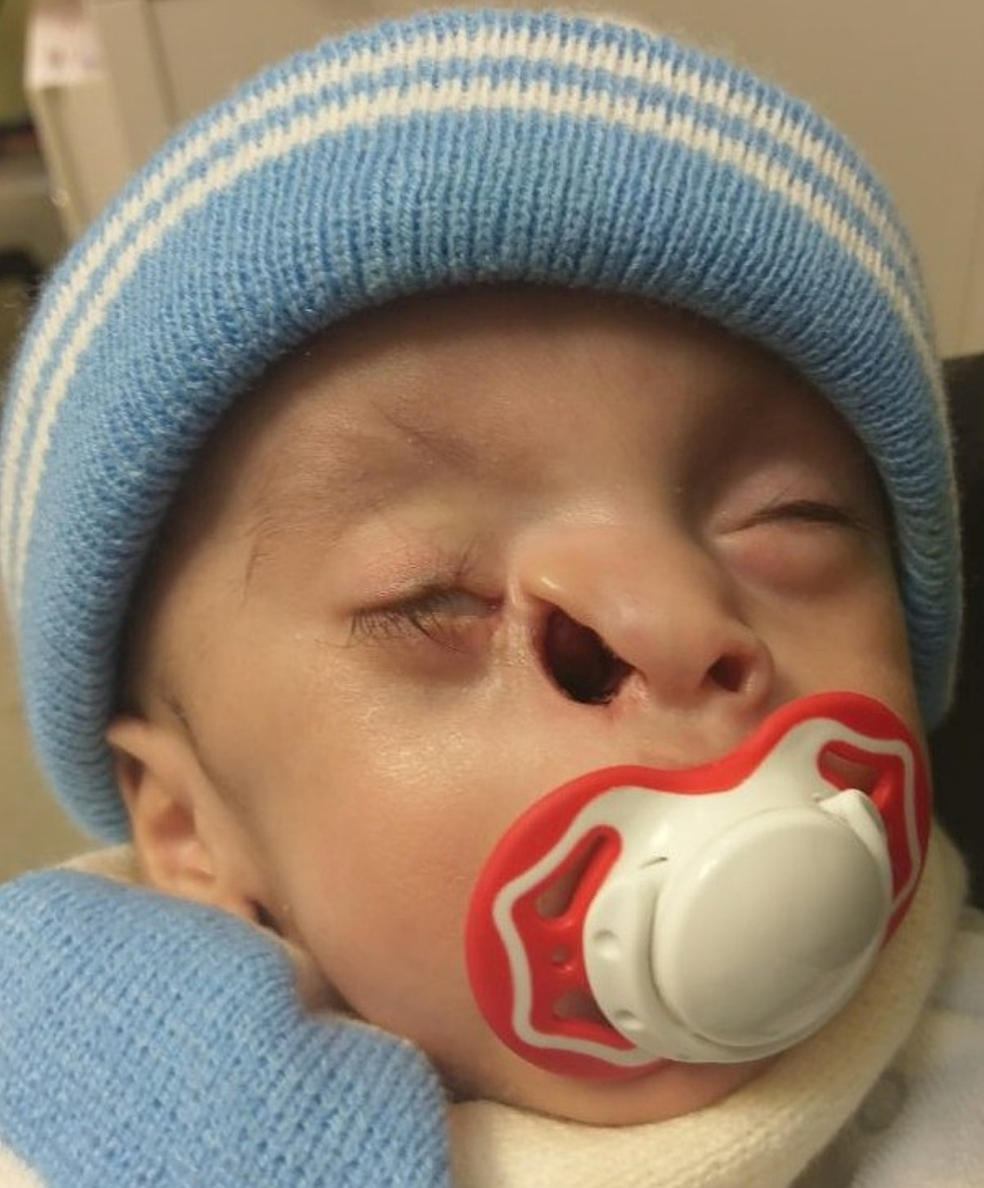
Right paramedian facial cleft and retraction of the right ala.

The examination revealed a febrile (39 ºC), pre-term male neonate, in evident respiratory distress, with a respiratory rate of 60 cycles per minute, noisy breathing, chest retraction, and Sp02 of 70% in ambient air, maintained at 92% with the use of an 8-liter non-rebreather face mask. Examination of the eyes showed hypertelorism, downward deviation and slanting of the right eye, proptosis, and difficulty closing the eyes. Nasal examination showed a retracted right ala and a large volume of nasal secretion (Figure 1). Attempts to pass an 8-Fr suction catheter tube met resistance 4 cm into the nasal cavity, and fiberoptic endoscopic examination showed bilateral CA. Oral examination showed that the patient was a mouth breather, with a retracted upper lip and a high arched palate. 

**Figure 2:**
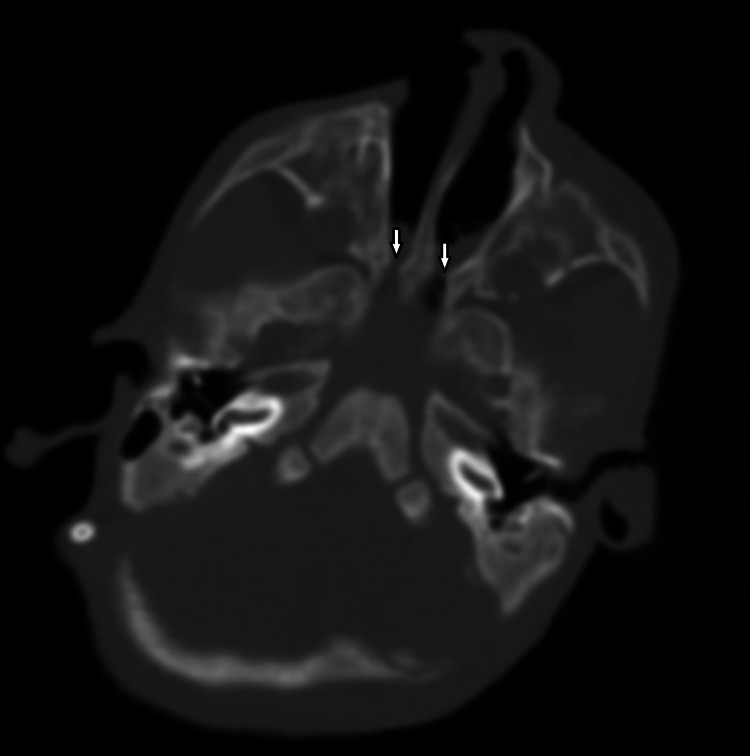
Axial computed tomography (bone window) of nose and paranasal sinuses showing bilateral choanal atresia (arrows).

**Figure 3:**
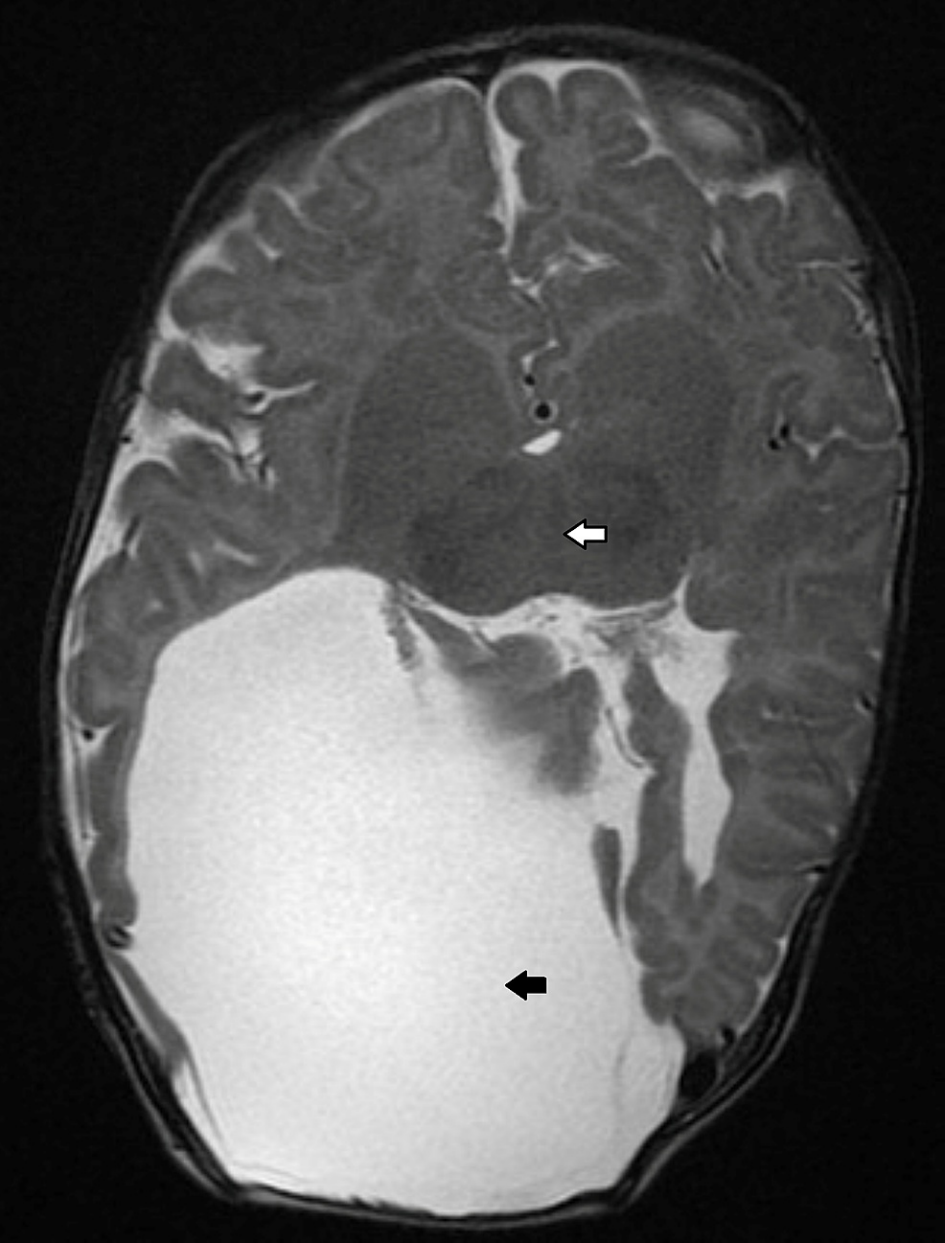
Axial section through cranial T2 MRI showing holoprosencephaly (white arrow) and schizencephaly (black arrow).

Ear examination showed no preauricular skin lesions (tags or pits) or deformity in the pinna or tympanic membrane. However, the ear retained its normal shape, but the left ear appearing larger than the right ear. Computed tomography (CT) of the paranasal sinuses revealed bilateral mixed type CA (Figure 2). Additionally, an MRI of the brain was performed on the same hospital admission. The findings were consistent with holoprosencephaly and schizencephaly (Figure 3).

The patient underwent surgery to repair the CA at a weight of 3.2 kg. He was laid in a supine position, pre-oxygenated, and intubated. The throat was then packed with wet gauze. Shoulder support was placed, and the head stabilized on a head-ring. Head dressing was applied, and the rest of the body was covered with a sterile drape.

**Figure 4:**
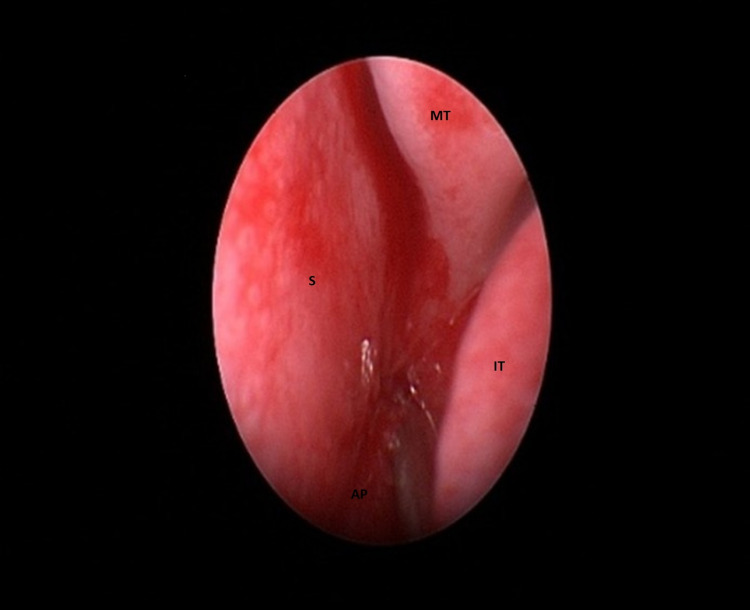
Endoscopic view of left choanal atresia before repair. AP = atretic plate, IT = inferior turbinate, MT = middle turbinate, S = septum.

**Figure 5:**
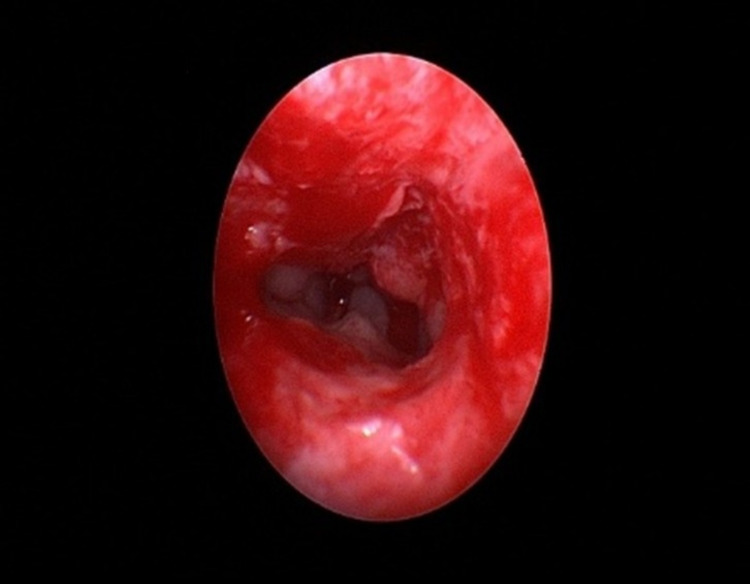
Endoscopic view showing an adequate choanal opening following endoscopic surgery.

The nasal cavities were prepared using drops of 0.5% xylometazoline for decongestion. The bilateral CA was approached via the endoscopic transnasal route. The bilateral atresia was gently punctured with a sickle knife, and an incision was performed in the medio-inferior part of the posterior choana. The flap was elevated successfully, the vomer removed, and the bony boundaries (pterygoid) were widened laterally using a curette. Adequate bilateral choanal openings were created (Figures 4, 5). 

The patient had an uneventful recovery postoperatively and was discharged after a few days. At the three-week follow-up, he gained weight (4.6 kg), and his symptoms improved. Six weeks after surgery, he demonstrated adequate choanal openings bilaterally, no further episodes of nasal obstruction, no difficulties in breathing or feeding, and further weight gain (5.5 kg). 

## Discussion

CA occurs more commonly in females than in males (2:1); moreover, unilateral atresia (frequently on the right side) is more common than bilateral atresia [5-6]. The percentage of bony occlusion is 90%, and the remaining 10% comprises a membranous type of occlusion [7]. However, the modern workup of CT of the nose and paranasal sinuses showed a 30% pure bone atresia, 70% mixed membranous and bone atresia with no pure membranous atresia present [8-9].

Four theories have been proposed regarding the embryological origin of congenital CA; however, none has been proven. These theories include (1) persistence of the nasobuccal membrane of Hochstetter; (2) persistence of the buccopharyngeal membrane from the foregut; (3) abnormal location of the mesoderm forming adhesions in the nasal choanae, and (4) a misdirection of neural crest cell migration [3-10]. All these events occur between the fourth and eleventh gestational weeks [10-11].

Approximately 50% of patients have isolated malformations; associations with other congenital pathologies have been observed in coloboma, heart disease, CA, retarded development, genital hypoplasia, and ear anomalies (CHARGE) syndrome, including hypoplasia of the external ear and hearing loss) and other syndromes [6-12]. Several associated defects have been described in the literature (Table 1) [13]. None of these defects were seen in the index case; however, the patient had a paramedian facial cleft, craniofacial anomalies, and congenital heart disease.

**Table 1:** Posterior choanal atresia and associated defects.

	Associated defects
Clefts	Lip, palate, nasal, facial, hypertelorism
Skull	Lowered skull base, microcephaly, cleidocranial dysostosis, craniosynostoses
CNS	Microcephaly, meningocele, retarded mental development, facial palsy, absent olfactory bulbs
Ear	Pinna anomalies, hearing loss, ossicular and cochleovestibular anomalies, aural atresia
Eye	Ocular colobomas, posterior embryotoxon, microphthalmia
Face	Micrognathia, mandibulofacial dysostosis, branchial remnants and anomalies, hypoplastic orbit and malar bone
Nose	Absent septum, nasolacrimal defects
Mouth and pharynx	Tongue abnormalities, macroglossia, nasopharyngeal defects
Larynx	Pharyngolaryngeal web
Cardiac and Respiratory	Atrial septal defect, patent ductus arteriosis, Tetrology of Fallot
Gastrointestinal	T-E fistula, ilial atresia, imperforate anus
Genitourinary	Hydronephrosis, hypogenitalism (males), duplication of upper urinary tract
Skeletal	Anomalous ribs, digital abnormalities, short neck, thoracic phocomelia
Syndromes	CHARGE syndrome, Apert syndrome, Treacher collins syndrome, VATER syndrome, Robin sequence, DiGeorge syndrome, Kallman syndrome.
Others	Hiatal and diaphragmatic hernias, absent spleen, minor chromosome abnormalities

The signs and symptoms of CA are many, varying in onset and severity depending on whether the deformity is unilateral or bilateral. Subjects with bilateral atresia present early with dyspnea and recurrent cyanosis and improve with crying, whereas subjects with a unilateral defect present with unilateral nasal obstruction and rhinorrhea [8]. Although unilateral CA may remain undiagnosed, bilateral CA usually is identified soon after birth and typically require immediate management [6]. Our patient presented to us with cyanosis around the mouth, respiratory distress, and poor feeding.

The clinical evaluation of CA by history and physical examination is essential to establish the diagnosis and rule out other anomalies and defects. CA is usually diagnosed by failure to pass a 6- or 8-Fr suction catheter through the nasal cavity into the nasopharynx; it should be noted that the distance from the nares to the posterior choana is at least 2.5 cm [5]. In the case reported, the anomaly was suspected when there was a failure to pass an 8-Fr suction catheter through the nasal cavity more than 4 cm from the ala of the nose. Rigid or flexible endoscopy can be used to visualize the atretic plate directly. Confirmation of the diagnosis is achieved with CT of the nasal cavity and paranasal sinuses, which provides insight into the etiology and thickness of the atresia [5]. These two diagnostic modalities were used for this patient.

Surgical repair is the gold standard treatment for CA. Various surgical techniques have been described, including the transnasal, transpalatal, and transeptal approaches, external rhinoplasty, or endoscopic routes. Endoscopic transnasal choanoplasty is the method most widely used [14-15]. Historically, the transpalatal approach was the first one described [7-16]. In our index case, the endoscopic transnasal approach was adopted. We used both soft tissue resection and bone curettage during the surgical treatment.

Restenosis has been reported to be the primary complication of the transnasal endoscopic approach. Approximately 9-36% of all patients may have restenosis after repair [7]. Injuries of the cavernous sinus, spinal canal, and cerebrospinal fluid leakage are the most dangerous surgical complications. These events are minimized by an advanced endoscopic approach that offers a direct visualization to the atretic plate and reduces intraoperative bleeding [17]. In our patient, we encountered no complications.

## Conclusions

Bilateral CA is usually diagnosed early because it is considered being incompatible with life. Mucoid or watery rhinorrhea, respiratory distress, asphyxia, and difficulty with feeding, that lasts from early few days of life without responding to any medical treatment, should alert the physician to consider bilateral CA and to perform endoscopic and CT examination at an early age. Early surgical intervention in the first week of life is necessary for survival. Despite being an emergency, with most cases corrected in the first week of life, there are few reported cases in the literature with a delayed diagnosis. This finding raises the question of whether all newborns are obligate nasal breathers or not.
